# Exploring Emotional, Restrained, and External Eating Behaviors: Impacts on Energy and Nutrient Intakes Among Korean Adults

**DOI:** 10.3390/nu17030473

**Published:** 2025-01-28

**Authors:** Geum-Bi Ryu, Young-Ran Heo

**Affiliations:** 1 Department of Food and Nutrition, Chonnam National University Graduate School, Gwangju 61186, Republic of Korea; rgb770@daum.net; 2 Division of Food and Nutrition and Research Institute for Human Ecology, Chonnam National University, Gwangju 61186, Republic of Korea

**Keywords:** eating behavior, energy intake, nutrient intake, adult, nutrition

## Abstract

Background/Objectives: This study aimed to investigate the levels of emotional, restrained, and external eating behaviors (EBs) among adults, categorize them, and analyze their energy and nutrient intake. Methods: A self-reported survey was administered to 522 Korean adults aged 19–64 years to evaluate their emotional, restrained, and external EBs. They were categorized into five types: Non-specific (NS), Emotional (Emo), Restrained (Res), External (Ext), and Combined (Com) EB types. Subsequently, energy intake, intake ratios and levels, and vitamin and mineral intakes were compared after adjusting for sex and age. Results: The Ext type participants consumed energy and an average of 3003.01 kcal per day, while those with Res type consumed 2415.77 kcal. Notably, both the Ext and Com types had higher proportions of excessive energy intake, while the NS and Res types displayed higher proportions of insufficient energy intake. The Emo type yielded a high ratio of lipid intake, while the Com type exhibited high ratios of both lipid and protein intakes. Furthermore, the Res type demonstrated lower vitamin E, niacin, and potassium intakes than the other types. Conclusions: Since the Ext type suggests excessive energy intake, and the Res type suggest intake of vitamin E, niacin, and potassium, identifying EB types provides a novel perspective for nutritional improvement strategies.

## 1. Introduction

Eating behavior (EB) in humans extends beyond the mere consumption of food for sustenance; it is influenced by various social, economic, cultural, and emotional factors. This complex and essential behavior significantly impacts both physical and mental health [1]. The factors influencing EB can be categorized into three forms: emotional, external, and restrained EBs, depending on the context wherein an individual consumes food. First, emotional EB view food consumption as a means of alleviating or coping with negative emotions. Second, external EB is characterized by food intake that is influenced by external stimuli, such as the appearance, taste, and aroma of food; the eating environment; and the presence of others, often disregarding internal signals like hunger. Finally, restrained EB refers to the conscious effort to regulate food intake based on weight or body shape [2].

EB can offer psychological stability, sensory satisfaction, and fulfillment of social needs when maintained at an appropriate level. However, when certain EBs become excessive, they potentially lead to health issues, including nutritional imbalances, which are influenced by these characteristics. Previous studies have indicated that emotional and external EBs are linked to binge eating, obesity, eating addiction, and metabolic syndrome [3,4,5]. Additionally, studies focusing on female college students, middle-aged women, and young adults have revealed that individuals with elevated stress and depression levels, along with poor emotional regulation skills, are more likely to engage in emotional eating [6,7,8]. Research has demonstrated that individuals who exhibit a high propensity for emotional eating tend to favor energy-dense foods rich in sugar and fat in their quest for immediate emotional relief or stimulation [9]. Similarly, those who practice external eating reportedly consume more food than necessary, even in the absence of hunger, thereby contributing to obesity [3]. Notwithstanding, while it seems desirable for individuals practicing restrained eating to regulate their food intake considering their body shape and weight, excessive moderation can lead to fixation on appearance and biases regarding weight, potentially eliciting eating disorders, such as anorexia nervosa and bulimia [10]. Additionally, college students who engage in restrained eating tend to experience greater dissatisfaction with their bodies and less dietary diversity [11].

Previous studies have suggested that health issues may arise depending on the degree of single EB characteristic among specific population groups, such as women and college students [3,4,5,6,7,8,9,10,11]. In addition, since humans are influenced by multiple variables when selecting what to eat [1], the types and amounts of food consumed are expected to vary depending on the factors exerting a greater impact in a given environment, that is, which EB characteristics are the strongest. In other words, the quantity of energy consumed and nutritional quality are anticipated to vary with the type of EB. However, considerably few studies have analyzed the differences in energy and nutrient intakes based on a comprehensive concept of EB in the general population [3]. Accordingly, we tried to propose the categorization of EBs in this study. We hypothesized that individuals might have a dominant trait of emotional, restrained, or external EBs, or that several traits might coexist strongly, or that they might simply have neutral eating habits. Rather than merely comparing differences between individuals who score above or below certain thresholds for a specific EB, we sought to explore the significance of integrated EB characteristics on nutritional status by using their distribution within the population. Thus, this study was conceived against the background of the lack of comprehensive information on EBs and nutritional status in Korean adults, as well as the limited number of studies using consistent indicators.

This study aimed to assess the degree of three EBs among Korean adults, categorize them, and compare the differences in energy and nutrient intakes. Ultimately, we sought to provide information revealing potential risks associated with adult EB types in terms of nutritional and health issues. Furthermore, by identifying individual EB types, we endeavored to uncover nutritional challenges that potentially form the basis for psychiatric eating disorders, obesity, and metabolic syndrome, as well as assist in formulating strategies for health improvement based on diverse EB types.

## 2. Materials and Methods

### 2.1. Participants

**Figure 1:**
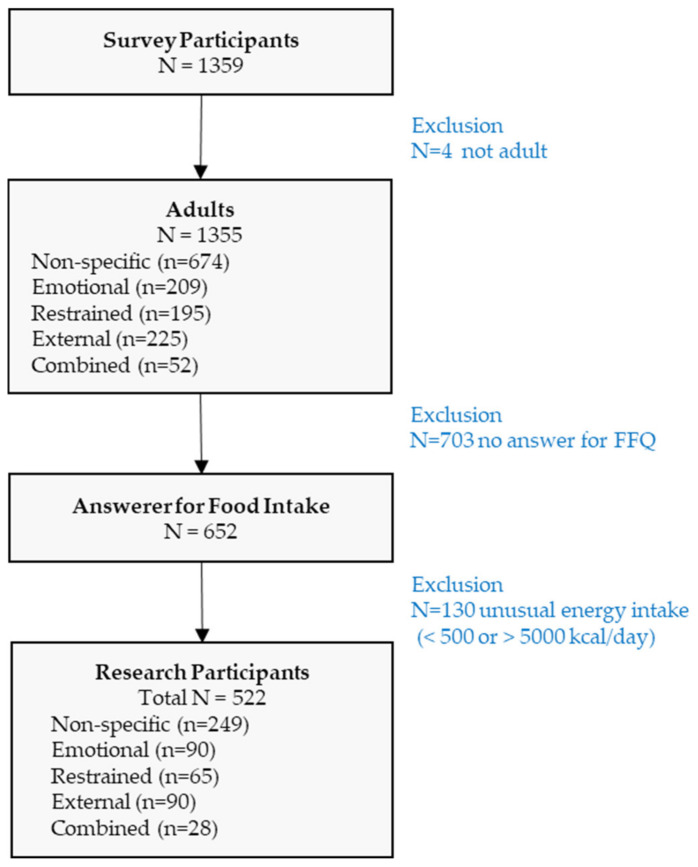
Inclusion process of the research participants.

This study’s participants were Korean adults aged 19–64 years. A stratified sampling was conducted with the aim of recruiting 200 men and 200 women, respectively, aged 19–39, 40s, and 50–64, by sex and age group. Participants were recruited nationwide through online promotions from June to November 2023, and only those who voluntarily agreed to participate were included in the online self-reported survey. As illustrated in Figure 1, a total of 1359 individuals participated in the survey; however, four individuals did not satisfy the age criteria, and 703 who did not complete the Food Intake Frequency Questionnaire (FFQ) were excluded. Additionally, the energy intake of 652 participants was calculated, and 130 individuals who <500 or >5000 kcal per day were excluded. Finally, data from 522 participants (140 men and 382 women) were analyzed.

### 2.2. Survey Contents

The survey included questions on sex, age, EB, and food intake to estimate energy and nutrient intakes.

#### 2.2.1. EB

The Dutch Eating Behavior Questionnaire (DEBQ) was utilized to examine the participants’ EBs. It was developed by Van Strien et al. in 1986 [2] and translated into Korean and its validity and reliability were confirmed by Kim et al. in 1996 [12]. The DEBQ is an interval scale comprising 32 questions, with responses ranging from 1 (strongly disagree) to 5 (strongly agree). It encompasses three distinct concepts. First, the questionnaire includes 10 items (nos. 1–10) that assess restrained EB, focusing on the control of food quantity and type, as well as meal timing, in relation to body weight and shape. Second, 13 items (nos. 11–23) evaluate emotional EBs, specifically measuring the desire to eat when feeling depressed, nervous, anxious, or disappointed. Finally, nine items (nos. 24–32) address external EBs by determining whether the taste or aroma of food, dining environment, or sight of others eating influences the urge to eat. To verify the validity of the DEBQ, exploratory factor analysis was conducted. One item (no. 1) with a commonality index < 0.5 and five items (nos. 4, 12, 28, 30, and 32) exhibiting a factor loading value < 0.4 were excluded from the analysis. The excluded questions were one item of emotional EB, “I want to eat when I’m bored”, two items of restrained EB, “I eat less when I gain weight”, “I know exactly what I’m eating”, and three items of external eating, “I want to buy something to eat when I pass the store”, “I can endure it without eating delicious food”, and “I eat food while preparing the meal”. Finally, the loading values for each item were higher than 0.6 for each single subdomain, lower than 0.3 for other subdomains, and all commonality indices were higher than 0.5. Following factor reduction, the Cronbach alpha values for the three EBs ranged from 0.855 to 0.957.

To classify EB types using three distinct EB scores, each score was divided into quartiles. The upper 25% cutoff points for emotional, external, and restrained EBs were 34.15, 27.69, and 22.62 points or higher, respectively. Participants in the top 25% of the emotional, restrained, and external EB scores were defined as emotional (Emo), restrained (Res), and external (Ext) types, respectively. When two EBs fell within the top 25%, classification was determined by comparing their similarities via cluster analysis. We check the dendrogram between groups through hierarchical cluster analysis and determined it to be divided into four main branches, cases not included. Subsequently, the data calculated into 5 clusters through K-means cluster analysis were compared with the quartile data of the three EBs. Consequently, if a participant exhibited both emotional and restrained EBs, they were classified as Emo. If a participant displayed both restrained and external EBs, they were classified as Res. In cases where both emotional and external EBs were present, the participant was classified as Ext. Finally, participants in whom all three EBs fell in the top 25% were designated as combined (Com), while those with none of the EBs in the top 25% were considered to exhibit non-specific (NS) EB type.

#### 2.2.2. Energy and Nutrient Intakes

A semi-quantitative food frequency questionnaire designed for the Korean population by Kim et al. in 2018 [13], with certain modifications, was employed to assess the participants’ energy and nutrient intakes. The FFQ in the reference was validated through an analysis of its correlation with the 3-day food record methods. The average correlation coefficient for calorie and macro nutrient intakes between the two tools was 0.402, which was significant. The questionnaire included the following 74 food items: grains and starches (11 items); meat, poultry, and eggs (10 items); fish and shellfish (12 items); beans and tofu (4 items); kimchi (3 items); fruits (14 items); milk and dairy products (4 items); fats and sugars (2 items); bakery items and nuts (4 items); tea and beverages (5 items); and alcoholic beverages (5 items). The investigation focused on the average single-time intake amount and annual intake frequency of each food item. The average serving size was classified into three categories: “0.5 times, 1 time, and 1.5 times”, based on the standard serving sizes recommended by prior research [13] and the Computer Aided Nutritional Analysis Program 5.0 (CAN-Pro 5.0, The Korean Nutrition Society, Seoul, Korea). Additionally, food consumption frequency was categorized as “almost never eaten”, “about once a month”, “2–3 times a month”, “1–3 times a week”, “4–6 times a week”, “about once a day”, and “more than twice a day”. In this study, the frequency, which was asked in the reference “1–2 times, 3–4 times, 5–6 times per week” and “1 time, 2 times, 3 time per day” was modified. Energy and nutrient intakes were calculated from the food intake data using CAN-Pro 5.0.

### 2.3. Statistical Analysis

Differences in EB scores based on the participants’ sex and age were analyzed using Student’s *t*-test and analysis of variance (ANOVA). Cluster analysis was used to classify EB types. Distribution differences in EB types according to sex and age as well as the energy intake status associated with these EB types were examined using Pearson’s chi-squared test. Furthermore, analysis of covariance (ANCOVA) was employed to evaluate energy intake according to EB type while controlling for sex and age. Additionally, the energy intake ratio and nutrient intake were assessed after adjusting for sex, age, and energy intake. A significance level of *p* < 0.05 was established for all statistical analyses, which were conducted using IBM SPSS Statistics (version 25.0; IBM Corp., Armonk, NY, USA).

## 3. Results

### 3.1. Sex- and Age-Related Differences in EB Among Korean Adults

#### 3.1.1. Sex- and Age-Related Differences in EB Scores Among Korean Adults

The participants’ EB scores were compared by sex and age (Table 1), revealing significant differences in EB scores attributable to both sex and age. Female participants exhibited notably higher emotional, restrained, and external EB scores than male participants. In terms of age, individuals in their 40s yielded higher emotional EB scores than those in their 50s and 60s as well as higher restrained EB scores than those in their 30s. Furthermore, participants in their 30s and 40s recorded higher external EB scores than those in their 50s or above.

#### 3.1.2. Sex- and Age-Related Differences in the Distribution of EB Types Among Korean Adults

The distribution of EB types according to participant sex and age is presented in Table 2. The NS type emerged as the most prevalent EB type among the participants, accounting for 249 individuals (47.7%). This was followed by the Emo and Ext types, each comprising 90 individuals (17.2%); the Res type with 65 individuals (12.5%); and the Com type with 28 individuals (5.4%). Furthermore, the distribution of EB types significantly varied with sex. Male participants exhibited a higher proportion of the NS type, whereas female participants demonstrated a greater prevalence of the Res, Ext, and Com EB types.

### 3.2. Difference in Energy Intake by EB Type Among Korean Adults

**Figure 2:**
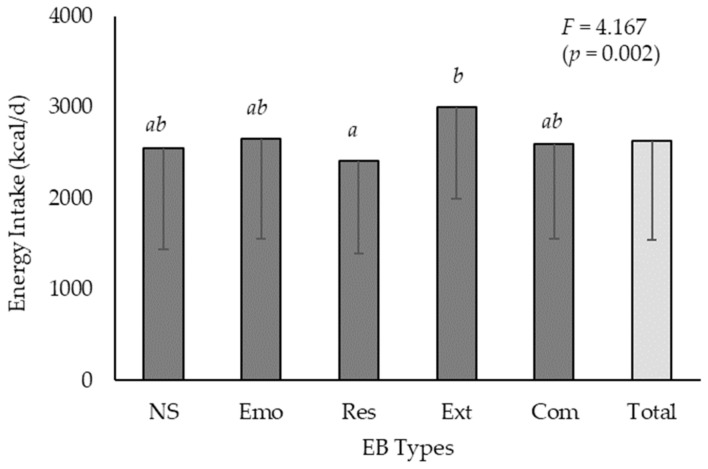
Difference in energy intake by EB type of Korean adults. The data were presented as the mean ± standard deviation. An analysis of covariance (ANCOVA) was conducted, adjusting for sex and age, followed by Scheffé’s post hoc test, which indicated a significant difference in the order of means (a < b).

The participants’ energy intake was evaluated according to EB type (Figure 2). The average daily energy intake for all participants was 2630.84 ± 1084.46 kcal. When analyzed by EB type, the energy intake was as follows: 2546.47 ± 1102.18, 2659.65 ± 1098.70, 2415.77 ± 1022.61, 3003.01 ± 1009.91, and 2591.67 ± 1035.58 kcal for the NS, Emo, Res, Ext, and Com type, respectively. After adjusting for sex and age, the differences in energy intake among the EB types were found to be statistically significant, with Ext type participants exhibiting a higher energy intake than Res type participants.

### 3.3. Distribution of Energy Intake Levels by EB Type Among Korean Adults

The distribution of energy intake levels by EB type is presented in Table 3. Participants were classified as having a “sufficient” energy intake when their consumption ranged from 75% to 125% of the estimated energy requirement (EER), as defined by the 2020 Korean Dietary Reference Intakes, stratified by sex and age. An intake < 75% of the EER was classified as “deficient”, while that > 125% of the EER was classified as “excessive”. Among the 522 participants analyzed, 177 (33.9%), 82 (15.7%), and 263 (50.4%) were classified as sufficient, deficient, and excessive energy consumers, respectively. The differences in energy intake levels across the various EB types were found to be statistically significant. Notably, the NS and Res types exhibited a higher prevalence of energy deficiency, while the Ext and Com types demonstrated a greater incidence of excessive energy intake relative to the average value.

### 3.4. Energy Intake Ratio by EB Type Among Korean Adults

The variations in energy intake ratio by EB types are presented in Table 4. The carbohydrate, lipid, and protein proportions of the total energy intake among participants were 59.77 ± 10.42%, 24.44 ± 8.46%, and 14.52 ± 2.88%, respectively. These values fall within the acceptable macronutrient distribution range of carbohydrates, lipids, and proteins, that is 55–65%, 15–30%, and 7–20%, respectively. Furthermore, a significant difference in energy intake ratios was observed across the EB types after controlling for the participants’ sex, age, and total energy intake. Specifically, the carbohydrate proportion was higher in the NS and Ext types than in the Com type. Conversely, the lipid proportion was higher in the Emo and Com types relative to the NS type. Finally, the protein proportion was greater in the Com type than in the Ext type.

### 3.5. Differences in Nutrient Intake by EB Type of Korean Adults

The differences in major nutrient intake by EB type are presented in Table 5. After adjusting for sex, age, and total energy intake, significant differences in nutrient intake (carbohydrates, lipids, vitamin E, niacin, potassium, and copper) were observed across EB types. Specifically, carbohydrate intake was evidently higher in the Ext type than in all other types, while lipid intake was also higher in the Ext type than in the NS type. Furthermore, vitamin E intake was lower in the NS and Res types than in the Ext and Com types. Additionally, niacin intake was lower in the Res type than in the Com type, and potassium intake was lower in both the NS and Res types than in the Com type. However, the post hoc analysis indicated that copper intake did not exhibit any statistically significant differences.

## 4. Discussion

This study elucidates the disparities in energy and nutrient intakes among Korean adults, contingent upon their EB types. Initially, the analysis revealed variations in the distribution of EB scores and types in relation to sex and age. Therefore, total energy intake was assessed after adjusting for sex and age. The findings indicated that Ext type individuals consumed more energy than those with Res type. Furthermore, the proportion of people with excessive energy intake among Ext and Com types was notably higher than the average value. Conversely, the proportion of people with deficient energy intake among NS and Res types was higher than the average value. The Ext type is characterized by the selection of food and eating patterns that are influenced by external stimuli, rather than internal physiological signals, such as hunger or satiety. This behavior is posited to contribute to overeating, binge eating, and a lack of control over food intake, resulting in excessive energy consumption. Previous research has indicated that women exhibit higher levels of stress and neuroticism than men, alongside elevated rates of emotional and external eating. Additionally, women’s EBs have been found to strongly correlate with specific personality traits [14]. Moreover, a study involving twins and their families suggested that symptoms of depression and anxiety significantly impact emotional and external eating, with these behaviors being associated with genetic factors. In contrast, restrained eating were evidently influenced by environmental factors [15]. This study partially corroborates earlier findings regarding the sex-related differences in EBs. Considering that both genetic and environmental factors apparently play a role in shaping EBs, the results suggest the potential of leveraging an understanding of EB types as a strategic intervention for promoting appropriate energy intake. Intervention strategies for overweight and obese individuals with emotional eating characteristics have reported psychological approaches based on mindfulness and behavioral treatments that coordinated diet and physical activity [16]. Among these interventions, the most effective cognitive behavior therapy focused on the importance of understanding and recognizing emotional eating characteristics by oneself. However, there are still widespread differences in these intervention methods and their effects, and previous researchers claim that a standardized tool for identifying EB and understanding the mechanisms that explain the relationship between EB and health issues is needed. In that regard, this study suggested a method for identifying comprehensive EB characteristics, and revealed how differences in energy and nutrient intake based on EB types might have provided a step toward understanding these mechanisms.

The analysis revealed significant variations in the energy intake ratio based on EB type. Specifically, the proportional contribution of carbohydrates to total energy intake was found to be greater among NS type individuals than among their Com type counterparts. Conversely, the proportional contribution of lipids was higher in both the Emo and Com types than in the NS type. Additionally, the protein proportion was elevated in the Com type relative to that in the Ext type. Considering the carbohydrate-centric dietary pattern prevalent among Koreans, the Emo type was observed to exhibit a higher lipid intake than other EB types, while the Com type demonstrated increased proportions of both lipids and proteins. These findings suggest that individuals with emotional eating tendencies tend to favor energy-dense foods that provide immediate emotional gratification [9]. Furthermore, the Com type, characterized by elevated levels of emotional, restrained, and external EBs, displayed excessive energy intake, with higher proportions of lipid and protein consumption compared with other types. This indicates a substantial overall food intake, implying a significant consumption of lipid and protein sources, such as fish, meat, oils, and/or nuts. High-fat diets and excessive energy intake contribute to disease such as obesity and metabolic syndrome [17], so in the case of Emo and Com types, they need to be aware of their own eating characteristics and pay attention to proper energy consumption. Through this study, we were able to categorize and compare various EBs, thereby enhancing our understanding of the characteristics associated with individual eating patterns, as well as the complexities of the Com type, which encompasses all three tendencies. Individuals within this category encounter challenges related to excessive energy intake. In light of these findings, we advocate for the development of a new definition of and further research into the Com type.

Nutrient consumption varies with individual EB type, with a particular emphasis on major nutrients, such as carbohydrate, lipid, vitamin E, niacin, and potassium. Notably, carbohydrate intake was significantly higher in the Ext type than in all other types, while lipid intake was also higher in the Ext type than in the NS type. This highlights the necessity for increased awareness regarding the risks associated with excessive energy intake, including overeating and binge eating, particularly within the Ext type. Given that the Ext type is more prevalent among women and individuals in their 20s and 30s, paying attention to and conducting further research on the eating environments and external factors influencing EBs in these demographic groups are imperative. A case–control study that analyzed the effects of mindful eating induction on food choices and energy intake in adult women showed that external eating functions as a moderator [18]. In other words, the mindful eating intervention group had reduced intakes of high-energy dense food and energy compared to the control group, and there was a greater reduction effect in those with high external eating levels. Therefore, these results suggest that cognitive and focused eating strategies might be useful for improving excessive energy intake related to external EB.

The consumption of vitamins, specifically vitamin E and niacin, varied with EB type. Notably, vitamin E intake was significantly lower in the Res and NS types than in the Ext and Com types. Overall, vitamin E intake was deemed adequate across all EB types, surpassing the recommended intake level of 12 mg for Korean adults aged 19–64 years. Vitamin E is recognized among the most effective antioxidants in the human body—found in sources such as vegetable oils, leafy greens, nuts, and dairy products—and serves a crucial role in antioxidant activity, blood metabolism, and skin health [19]. Therefore, individuals with lower intake levels, such as Res and NS EB types, may need to be more cautious to avoid vitamin E deficiency.

Niacin intake was lower in the Res type than the Com type. In particular, niacin consumption in the Res type was either inadequate or only marginally higher than the recommended intake for Korean adults, which is 16 mg for men and 14 mg for women. This situation necessitates caution to prevent potential deficiency. Symptoms associated with niacin deficiency, commonly known as pellagra, include dermatitis, weakness, diarrhea, sleep disturbances, and psychiatric disorders [20]. Furthermore, individuals with low niacin intake predictably possess an elevated risk of developing glaucoma [21,22]. Consequently, investigating health issues related to niacin deficiency, particularly symptoms of skin and ocular diseases, in adults who engage in restrained eating, such as extreme dieting, is paramount. The primary dietary sources of niacin include fish, meat, grains, beans, and nuts. Identifying the specific foods predominantly avoided by weight-sensitive Res type individuals and addressing dietary practices that may impede the intake of vitamin E and niacin would be beneficial.

Potassium intake was significantly lower in NS and Res type individuals than in the Com type. This intake was evidently below the adequate intake level established for Korean adults (3500 mg). Potassium is a critical nutrient that plays an essential role in various cellular functions, including maintaining fluid balance and osmotic pressure. A deficiency in dietary potassium, often owing to the inadequate consumption of foods such as fruits, vegetables, grains, dairy products, and meats, has been associated with hypertension and cardiovascular issues [23]. Therefore, individuals with restrained eating patterns must recognize the importance of managing body shape and weight via alterations to meal composition rather than merely restricting their diet. Not only that, previous studies have shown that children and adolescents with restrained EB and anorexia nervosa (AN) had a lower average intake of energy—especially fat—and whole vitamins and minerals such as calcium, potassium, phosphorus, and magnesium, and were at higher risk of lacking nutrient intake. Among the psychological factors, studies suggest that individuals with restrained EB or AN have dysfunctional beliefs and attitudes about food or nutrients, attempting energy restriction, mainly by reducing the consumption of foods with high fat content [24]. Therefore, a psychological approach is needed to correct beliefs and attitudes toward restrained eating along with nutritional interventions.

Finally, this study focused on the characteristics that influence an individual’s eating choices, which could help infer nutritional problems. Particularly, we categorized EB by analyzing emotional, restrained, and external EB characteristics, and also identified differences between non-specific and combined EB types. Therefore, analyzing EB might be useful as a new aid for nutritional assessment. Also, the findings of this study provide preliminary data that may elucidate the relationships between specific nutritional issues and eating-related diseases, taking into account demographic characteristics and the nature of EB types. However, as this examination is a preliminary study on the association between nutritional status and EB in Koreans, it has several limitations. First, in terms of research design, this study is a cross-sectional sample study, and data were collected only through self-reported surveys, so the response bias cannot be ignored. In addition, during the inclusion process, it is necessary to consider the participants who were excluded because they did not respond to FFQ. Moreover, for the participants excluded due to abnormal calorie intake, it was difficult to determine whether it was a blind spot in the self-reported survey or the result of actual intake. Therefore, the empirical and longitudinal studies using observational research methods or biological anthropometric analyses are needed in the future. In addition, in terms of contents, it is important to note that the survey conducted in this study neither examined nutritional supplement and functional food nor screened for special populations, such as pregnant or breastfeeding women, who may require additional nutrient supplementation. This limitation underscores the necessity for further research to address these gaps.

## 5. Conclusions

This study analyzed the differences in energy and nutrient intakes among Korean adults based on their EB. Considering that the distribution of EB scores and types varied with sex and age, appropriate adjustments were implemented. The findings reveal that energy intake is significantly higher in Ext type individuals than in their Res type counterparts. Furthermore, Ext and Com type individuals exhibit a greater tendency to exceed the recommended energy intake, whereas NS and Res type individuals are more prone to energy deficiency. Additionally, the consumption of carbohydrates, lipids, vitamin E, niacin, and potassium varied according to EB type. Ext type is associated with a higher risk of overeating, while Res type is associated with potential deficiencies in vitamin E, niacin, and potassium. This study’s findings are meaningful in that they provide quantitative data on energy and nutrient intakes; thus, they are anticipated to offer novel insights into the relationship between nutritional issues and the EB types. Continued research on EBs and health issues using various methods and populations is necessary to establish EB as a reliable nutritional indicator.

## Figures and Tables

**Table 1:** Sex- and age-related differences in EB scores among Korean adults.

Variable		EB Score		
		Emotional	Restrained	External
Total		25.36 ± 11.89 ^(1)^	22.14 ± 8.46	19.24 ± 5.06
Sex	Male	22.04 ± 11.18	20.09 ± 7.85	17.54 ± 5.19
	Female	26.58 ± 11.92	22.90 ± 8.56	19.87 ± 4.86
	*t* ^(2)^ *(p)*	−3.924 (0.000)	−3.400 (0.001)	−4.748 (0.000)
Age	19–29	26.33 ± 12.42 ^ab^	21.85 ± 8.80 ^ab^	19.44 ± 4.95 ^ab^
	30s	25.09 ± 11.67 ^ab^	21.05 ± 8.40 ^a^	19.64 ± 4.99 ^b^
	40s	27.01 ± 11.57 ^b^	24.63 ± 7.67 ^b^	19.63 ± 4.80 ^b^
	50–64	22.09 ± 11.07 ^a^	21.71 ± 8.31 ^ab^	17.76 ± 5.46 ^a^
	*F* ^(3)^ *(p)*	3.423 (0.017)	4.063 (0.007)	3.267 (0.021)

EB, eating behavior; ^(1)^ The data were presented as the mean ± standard deviation; ^(2)^ Student *t*-test; ^(3)^ ANOVA, Scheffé’s post hoc test, which indicated a significant difference in the order of means (a < b).

**Table 2:** Sex- and age-related differences in distribution of EB types among Korean adults.

Variable		EB Type					
		Total	NS	Emo	Res	Ext	Com
**Total**		522 (100) ^(1)^	249 (47.7)	90 (17.2)	65 (12.5)	90 (17.2)	28 (5.4)
Sex	Male	140 (100)	83 (59.3)	24 (17.1)	13 (9.3)	16 (11.4)	4 (2.9)
	Female	382 (100)	166 (43.5)	66 (17.3)	52 (13.6)	74 (19.4)	24 (6.3)
	χ^2 (2)^ *(p)*	12.914 (0.012)					
Age	19–29	174 (100)	80 (46.0)	30 (17.2)	19 (19.0)	33 (19.0)	12 (6.9)
	30s	153 (100)	75 (49.0)	23 (15.0)	16 (10.5)	33 (21.6)	6 (3.9)
	40s	104 (100)	42 (40.4)	24 (23.1)	18 (17.3)	13 (12.5)	7 (6.7)
	50–64	91 (100)	52 (57.1)	13 (14.3)	12 (13.2)	11 (12.1)	3 (3.3)
	χ^2^ *(p)*	15.968 (0.193)					

EB, eating behavior; NS, non-specific; Emo, emotional; Res, restrained; Ext, external; Com, combined; ^(1)^ The data were presented as a number (percentage); ^(2)^ Chi-Squared test.

**Table 3:** Distribution of energy intake levels by EB type among Korean Adults.

EB Type	Total	Energy Intake Level			χ^2 (2)^ *(p)*
		Deficient	Sufficient ^(1)^	Excessive	
Total	522 (100) ^(3)^	82 (15.7)	177 (33.9)	263 (50.4)	15.686 (0.047)
NS	249 (100)	47 (18.9)	87 (34.9)	115 (46.2)	
Emo	90 (100)	12 (13.3)	35 (38.9)	43 (47.8)	
Res	65 (100)	11 (16.9)	25 (38.5)	29 (44.6)	
Ext	90 (100)	8 (8.9)	21 (23.3)	61 (67.8)	
Com	28 (100)	4 (14.3)	9 (32.1)	15 (53.6)	

EB, eating behavior; NS, non-specific; Emo, emotional; Res, restrained; Ext, external; Com, combined; ^(1)^ Data were classified as “Sufficient” when it met 75% to 125% of the estimated energy requirements based on sex and age. It was categorized as “Deficient” if it fell below this range, and as “Excessive” if it exceeded this threshold; ^(2)^ Chi-Squared Test; ^(3)^ The data were presented as a number (percentage).

**Table 4:** Differences in energy intake ratio by EB type among Korean Adults.

EB Type	Energy Intake Ratio		
	Carbohydrate	Lipid	Protein
Total	59.77 ± 10.42 ^(1)^	24.44 ± 8.46	14.52 ± 2.88
NS	61.08 ± 10.52 ^c^	23.14 ± 8.54 ^a^	14.34 ± 2.97 ^ab^
Emo	56.96 ± 11.21 ^ab^	26.81 ± 8.87 ^b^	14.91 ± 3.07 ^ab^
Res	58.48 ± 10.74 ^abc^	25.39 ± 8.64 ^ab^	15.15 ± 2.87 ^ab^
Ext	60.86 ± 8.33 ^bc^	24.13 ± 6.92 ^ab^	13.90 ± 2.29 ^a^
Com	56.56 ± 10.13 ^a^	27.08 ± 8.52 ^b^	15.45 ± 2.55 ^b^
*F* ^(2)^ *(p)*	4.048 (0.003)	4.387 (0.002)	3.023 (0.018)

EB, eating behavior; NS, non-specific; Emo, emotional; Res, restrained; Ext, external; Com, combined; ^(1)^ The data were presented as the mean ± standard deviation; ^(2)^ The analysis employed ANCOVA, adjusting for sex, age, and total energy intake, followed by Duncan’s post hoc test, which indicated a significant difference in the order of means (a < b < c).

**Table 5:** Differences in nutrient intakes by EB type of Korean adults.

Nutrients		Total	EB Types					*F* ^(1)^ *(p)*
			NS	Emo	Res	Ext	Com	
Energy	Carbohydrate	393.1 ± 176.4 ^(2)^	388.4 ± 179.7 ^a^	378.9 ± 179.3 ^a^	354.7 ± 169.3 ^a^	455.3 ± 161.9 ^b^	369.1 ± 60.0 ^a^	2.887 (0.022)
	Lipid	71.6 ± 40.6	65.7 ± 39.9 ^a^	79.0 ± 43.8 ^ab^	68.2 ± 38.0 ^ab^	81.2 ± 38.7 ^b^	77.1 ± 40.0 ^ab^	3.063 (0.016)
	Protein	94.9 ± 43.7	91.0 ± 44.9	98.8 ± 47.2	89.8 ± 37.9	104.5 ± 40.4	99.0 ± 40.5	2.227 (0.065)
Vitamin	A	727.0 ± 615.6	701.8 ± 602.6	741.6 ± 584.9	688.0 ± 493.0	803.3 ± 773.4	749.7 ± 21.9	0.123 (0.974)
	D	4.4 ± 3.4	4.3 ± 3.8	4.6 ± 3.3	4.0 ± 2.4	4.6 ± 2.9	4.3 ± 3.1	0.385 (0.819)
	E	19.2 ± 9.9	18.0 ± 9.9 ^a^	20.2 ± 9.9 ^ab^	17.6 ± 7.6 ^a^	21.4 ± 9.9 ^b^	22.4 ± 12.3 ^b^	2.804 (0.025)
	K	173.9 ± 167.1	169.1 ± 146.1	170.7 ± 159.5	158.2 ± 133.5	198.1 ± 241.6	186.4 ± 145.6	0.160 (0.958)
	C	111.9 ± 110.5	107.3 ± 110.3	119.3 ± 125.0	109.5 ± 93.0	110.6 ± 99.0	139.7 ± 135.4	1.291 (0.272)
	Thiamine	2.5 ± 1.2	2.4 ± 1.2	2.6 ± 1.2	2.4 ± 1.1	2.8 ± 1.2	2.6 ± 1.0	0.875 (0.478)
	Riboflavin	2.1 ± 1.0	2.0 ± 1.1	2.2 ± 1.0	2.0 ± 1.0	2.3 ± 1.0	2.2 ± 0.9	1.389 (0.236)
	B6	2.8 ± 2.2	2.8 ± 2.4	3.0 ± 2.2	2.5 ± 1.3	2.9 ± 2.0	2.9 ± 2.1	1.169 (0.324)
	Niacin	16.4 ± 7.9	16.0 ± 8.1 ^ab^	16.8 ± 8.9 ^ab^	14.8 ± 6.0 ^a^	17.5 ± 6.8 ^ab^	18.3 ± 9.0 ^b^	2.997 (0.018)
	Folic acid	502.3 ± 230.2	482.3 ± 233.0	491.7 ± 229.6	483.2 ± 201.1	566.7 ± 221.3	552.5 ± 266.6	1.733 (0.141)
Mineral	Ca	513.2 ± 290.3	488.9 ± 289.2	499.5 ± 267.5	508.0 ± 269.9	592.6 ± 337.1	530.2 ± 217.0	0.669 (0.614)
	P	1398.5 ± 620.7	1344.8 ± 644.7	1419.7 ± 634.4	1354.8 ± 552.6	1539.4 ± 592.6	1457.4 ± 552.9	1.736 (0.141)
	Mg	99.1 ± 60.1	95.1 ± 60.1	102.1 ± 66.8	99.3 ± 54.8	102.6 ± 56.0	113.5 ± 62.5	1.917 (0.106)
	Na	3375.2 ± 1886.5	3170.3 ± 1853.3	3643.9 ± 2195.7	3052.4 ± 1450.1	3820.7 ± 1902.9	3650.9 ± 1639.9	1.897 (0.110)
	K	3078.5 ± 1495.6	2921.2 ± 1491.8 ^a^	3156.0 ± 1634.2 ^ab^	3010.2 ± 1231.7 ^a^	3354.7 ± 1431.5 ^ab^	3498.0 ± 1695.2 ^b^	2.682 (0.031)
	Fe	17.7 ± 8.8	17.24 ± 8.9	18.0 ± 9.8	16.6 ± 7.1	19.3 ± 9.0	18.7 ± 7.9	1.015 (0.399)
	Zn	13.3 ± 6.3	13.1 ± 6.5	13.6 ± 7.1	12.2 ± 5.1	14.6 ± 5.8	13.3 ± 6.2	1.048 (0.382)
	Cu	725.6 ± 409.0	693.1 ± 412.4	780.7 ± 483.3	705.5 ± 345.1	743.8 ± 343.5	826.3 ± 438.4	2.516 (0.041)
	Mn	2.6 ± 1.8	2.5 ± 1.9	2.6 ± 1.9	2.6 ± 1.6	2.6 ± 1.7	2.7 ± 1.5	1.146 (0.334)
	I	77.2 ± 49.3	72.9 ± 51.3	76.1 ± 41.9	79.7 ± 53.5	88.8 ± 47.2	76.2 ± 47.1	0.871 (0.481)
	Se	82.4 ± 46.6	77.1 ± 47.6	89.3 ± 53.0	78.3 ± 38.4	90.1 ± 39.2	92.9 ± 49.2	2.305 (0.057)

EB, eating behavior; NS, non-specific; Emo, emotional; Res, restrained; Ext, external; Com, combined; The units of measurement utilized for various nutrients are as follows: grams (g) for carbohydrates, lipids, and proteins; micrograms of Retinol Activity Equivalent (μg RAE) for Vitamin A; micrograms (μg) for Vitamins D and K, folic acid, copper (Cu), iodine (I), and selenium (Se); and milligrams (mg) for thiamine, riboflavin, Vitamin B6, niacin, calcium (Ca), phosphorus (P), magnesium (Mg), sodium (Na), potassium (K), iron (Fe), zinc (Zn), and manganese (Mn); ^(1)^ The analysis employed ANCOVA, adjusting for sex, age, and total energy intake, followed by a Duncan post hoc test (a < b); ^(2)^ The data were presented as the mean ± standard deviation.

## Data Availability

The data that support the findings of this study are not publicly available due to the data containing information that could compromise participant privacy but are available from the corresponding author on reasonable request.
